# Laparoscopic retrieval of sharp foreign body: An innovative approach

**DOI:** 10.1016/j.ijscr.2020.06.002

**Published:** 2020-06-11

**Authors:** P.O. Igwe, N.E. Brownson, S.L. Harcourt, N. Ejindu

**Affiliations:** a Colorectal and Minimal Access, General Surgery Unit, Department of Surgery, University of Port Harcourt Teaching Hospital (UPTH), Port Harcourt, Rivers State, Nigeria; b Orthopedic Surgery Unit, Department of Surgery, University of Port Harcourt Teaching Hospital (UPTH), Port Harcourt, Rivers State, Nigeria; c Radiology Department, University of Port Harcourt Teaching Hospital (UPTH), Port Harcourt, Rivers State, Nigeria

**Keywords:** Sharp foreign body, Laparoscopy, Innovative approach

## Abstract

**Background:**

Laparoscopy is a relatively new surgical approach in developing countries. Migrated foreign bodies into the body cavities could be very distressing to patients, relations and caregivers. There are paucity of migrated Steinmann pin and laparoscopic retrieval methods in literature.

**Aim:**

An innovative approach of laparoscopic retrieval of migrated sharp foreign body (Steinmann pin).

**Methods:**

A two-port technique was used to retrieve the pin. These ports consisted of optical supra-umbilical port (10 mm) and left iliac fossa port of 10 mm. They were aligned in the direction of sharp end of the foreign body, to aid retrieval of the Steinmann pin.

This procedure was performed on a 34-year-old man who presented with migration of Steinmann pin into abdominal cavity.

He had open reduction of old unreduced left posterior hip dislocation. A Steinmann pin with a diameter of 4.5 mm and length of 140 mm was passed through the greater trochanter to the acetabular roof. The protruding end of the pin ought to have been bent. Three weeks post-surgery, the pin migrated.

Physical examination revealed vague tenderness over supra pubic region. A hard pointed object was palpable in the right iliac fossa. Abdominal X-ray and ultrasound were suggestive but not definite for pin location.

**Results:**

A sharp Steinman pin was retrieved from the abdominal cavity using laparoscopy.

**Conclusion:**

Migration of Steinmann pin is unusual. Laparoscopy is an innovative approach to foreign body retrieval. This could help reduce the morbidity that may be accompanied by other modalities such as open approach.

## Highlights

• Intra-abdominal migration of Steinmann pin is unusual.
• Laparoscopic foreign body retrieval has been reported in literature, Steinmann pin migration into abdominal cavity and its laparoscopic retrieval is yet to be reported.
• It is ideal to bend protruding end of Steinmann pin during insertion to prevent migration.
• We used a two-port approach by minimal access (laparoscopy) to retrieve the pin done in emergency condition.

## 1 Introduction

Laparoscopy is new in developing countries. It has been used in rare and difficult disease conditions [1]. Foreign bodies especially sharp ones within body cavities could be very distressing to patients, relations and caregivers. There is paucity of retrieval methods of migrated Steinmann pin in literature. An innovative approach could be likened to the use of laparoscopy in retrieving foreign bodies. This work has been reported in line with the SCARE criteria [2].

## 2 Aim

An innovative approach of laparoscopic retrieval of migrated Steinmann pin (sharp foreign body).

## 3 Case report

A 34-year-old man presented with migration of Steinmann pin into the abdominal cavity 3 weeks post operation.

He underwent open reduction of old unreduced left posterior hip dislocation. The reduction was maintained by insertion of a 140 mm long by 4.5 mm in diameter Steinmann pin. The pin was passed through the greater trochanter to the acetabular roof. Three weeks post-surgery the pin migrated.

Initially, he was asymptomatic. However, on the 8-week post operation, he started complaining of vague lower abdominal pain. He had no history of haematuria, haematochezia, and change in bowel habit. Also, no history of nausea, vomiting or abdominal distension.

Physical examination revealed vague tenderness over supra pubic region. A hard pointed object was palpable in the right iliac fossa suggestive of the foreign body. The surgical incision site had healed primarily.

**Fig. 1:**
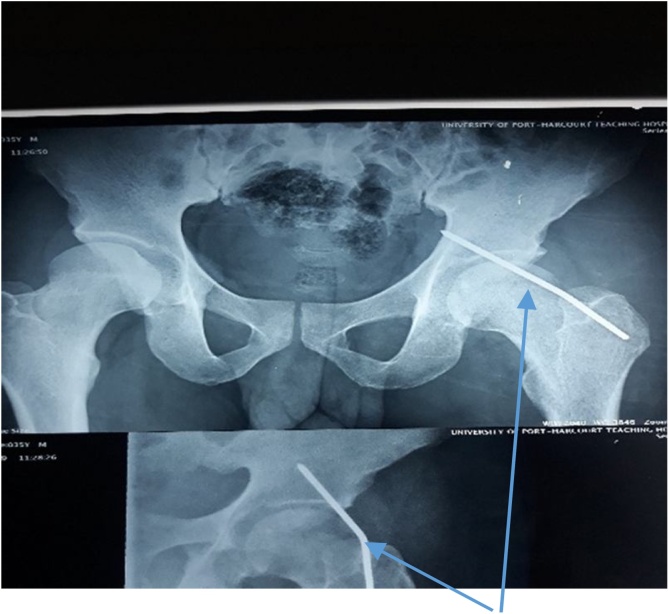
Steinmann pin 3rd week post pin insertion when pin was neither visible nor palpable at site of insertion (early onset of migration).

**Fig. 2:**
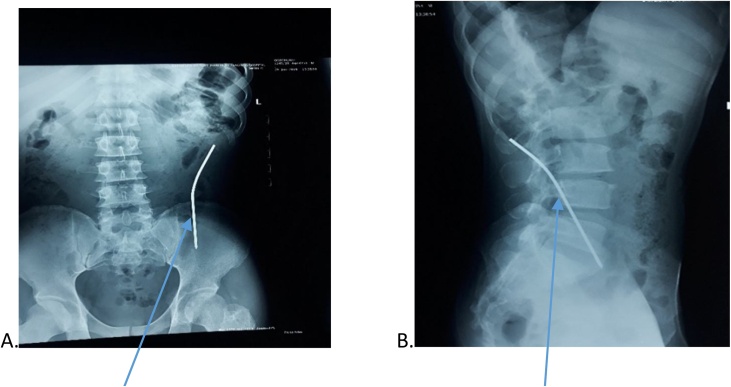
Plain abdominal radiographs showing migrated pin into abdominal cavity A) anteroposterior view. B) Lateral view.

**Fig. 3:**
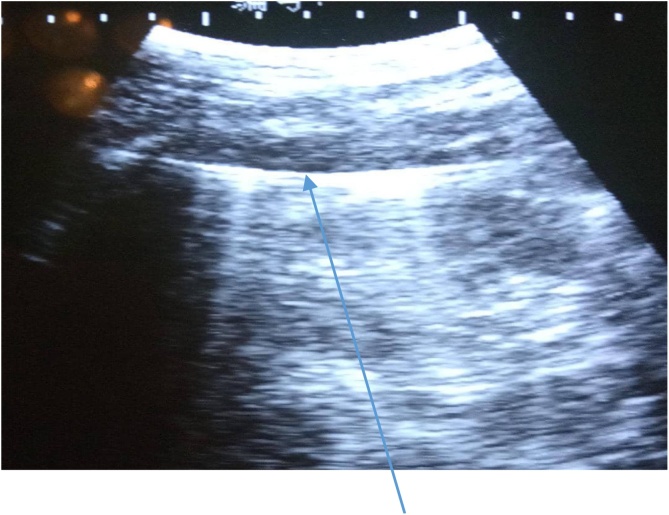
Two dimensional abdominal ultrasound scan image showing a curvilinear echogenic structure (Steinmann pin) with some posterior acoustic shadowing in the left lumbar region.

Investigations done showed that serum electrolyte, urea, creatinine and complete blood count were all within normal range. The abdominal ultrasound scan demonstrated a hyperechoic structure (Steinmann pin) with acoustic shadow. It could not localize the foreign body. Plain abdominal radiograph showed presence of pin. The pre-operative and post-operative radiographs at time of migration were as shown in Fig. 1, Fig. 2 respectively. The Fig. 1 showed Steinmann pin 3rd week post pin insertion (early onset of migration). The Fig. 2 showed migrated pin into abdominal cavity. (A) is the anteroposterior view, while (B) is the Lateral view. The Fig. 3 image showed two dimensional abdominal ultrasound scan of a curvilinear echogenic structure (Steinmann pin) with some posterior acoustic shadowing in the left lumbar region.

Patient was prepared and planned for emergency diagnostic and therapeutic laparoscopy.

## 4 Methods

**Fig. 4:**
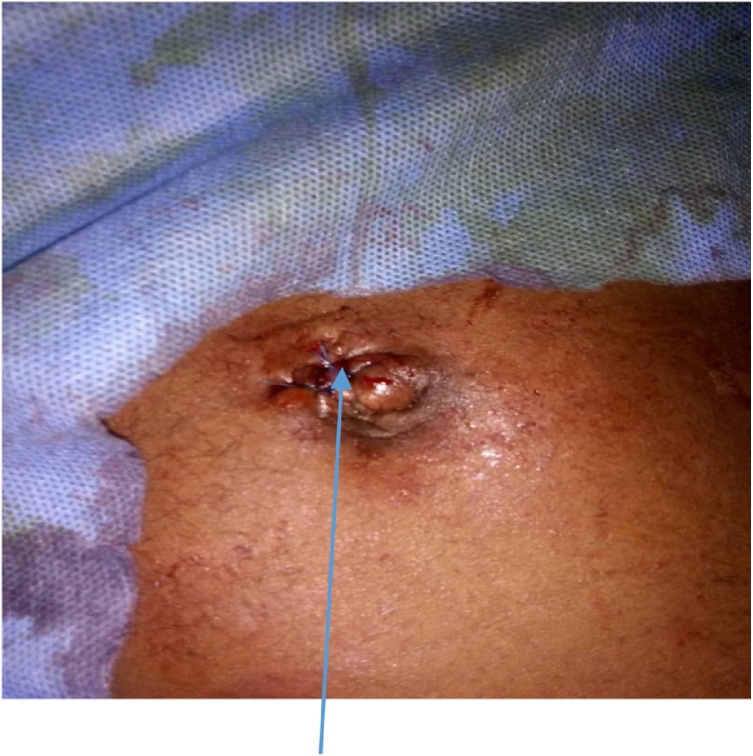
Supra-umbilical optical port.

A two-port laparoscopic technique was performed to retrieve the pin. The ports consisted of optical supra-umbilical port (10 mm) Fig. 4 and left iliac fossa port of 10 mm. They were aligned in direction of sharp end of the foreign body (Steinmann pin). The patient was wheeled directly from radiological suite to operating theatre maintaining same position. This was to prevent further migration of pin or visceral injury. The abdominopelvic ultrasonography was conducted in the operating room just before the operation.

General anaesthesia was used. The patient’s position was supine initially. Aseptic protocols were routinely observed. Supra-umbilical port was created using direct trocar access. Carbon dioxide gas was used to achieve pneumoperitoneum. The pre-set pressure was 12 mmHg. A 30° telescope was inserted and a diagnostic laparoscopy carried out. Intra-abdominal organs and bowel were essentially normal. The patient’s position was then changed to right lateral.

**Fig. 5:**
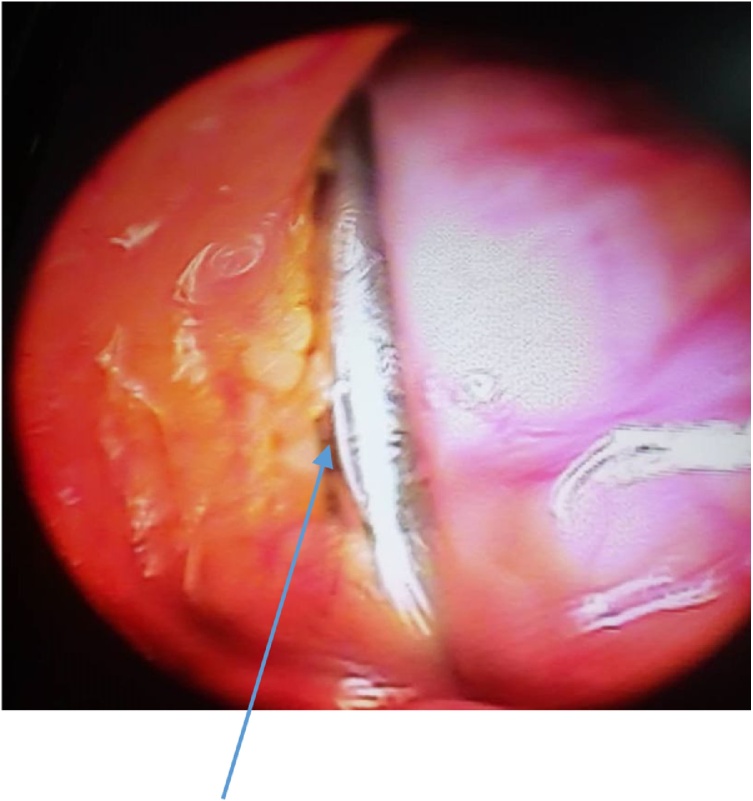
Pin in left paracolic gutter.

**Fig. 6:**
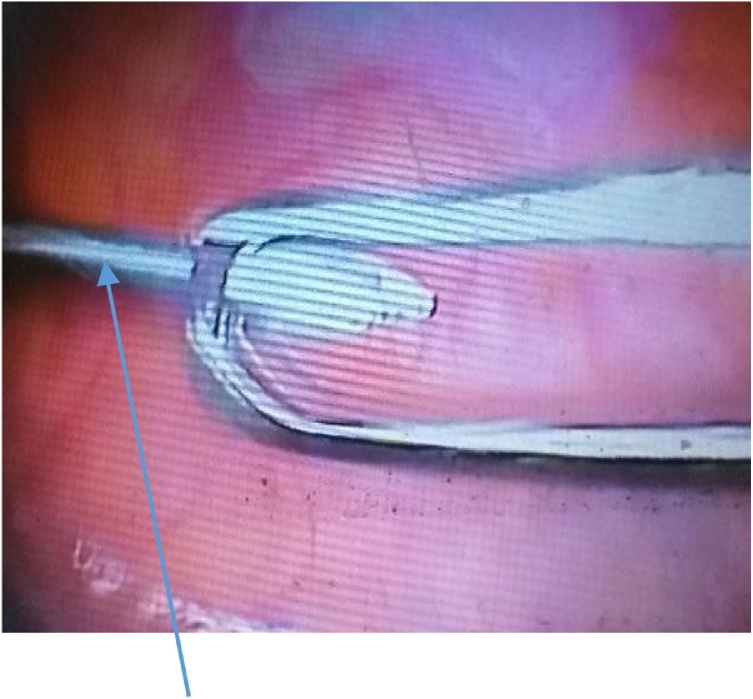
Pin during retrieval.

**Fig. 7:**
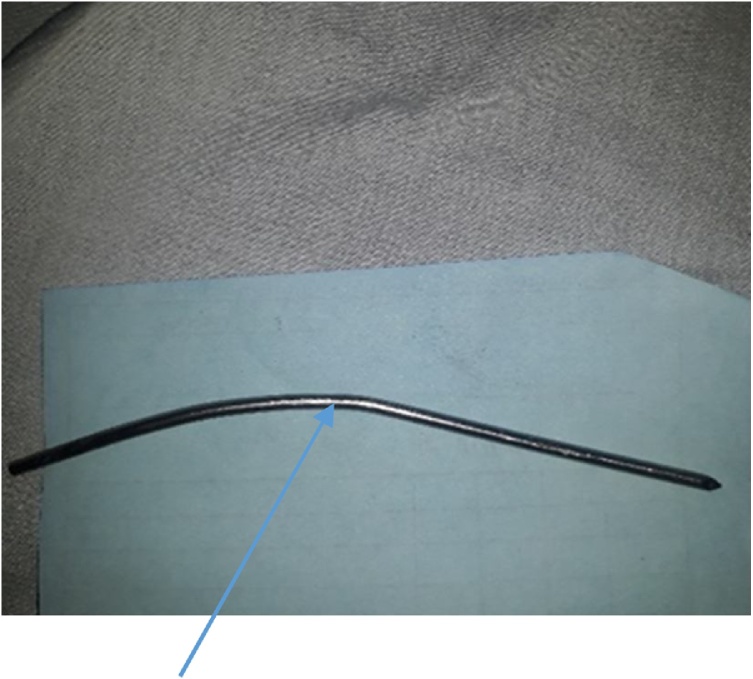
Pin after retrieval.

The Steinmann pin was seen intra-operatively, lying longitudinally in the left para-colic gutter. Fig. 5 A baseball diamond concept of triangulation was observed in inserting the secondary 10 mm port at the left iliac fossa. The left iliac fossa port was aligned in the direction of the sharp end of the pin to avert pin slippage injury during retrieval. The pin was removed via the left iliac fossa port under the direct vision of the telescope. A clawed forceps was used to retrieve the pin. There was no visceral injury observed. Fig. 6 showed pin grasped with claw forceps prior to retrieval, while picture of retrieved pin is shown in Fig. 7.

## 5 Results

A sharp Steinman pin measuring 4.5 mm in diameter and 140 mm in length was retrieved from the abdominal cavity. The visceral and other intra-abdominal structures were normal.

Patient was discharged 48 h post-operation, in a clinically satisfactory condition. His outpatient follow-ups were uneventful.

## 6 Discussion

The use of Steinmann pin after open reduction of old unreduced hip dislocation is a common practice in orthopaedic surgery [3].

There are reports showing Intra-abdominal migration of pins and wires after fixation of proximal femoral fractures and hip surgeries [4,5]. The pins are sharp ended on one side. The sharp ended part poses potential danger to visceral organs during migration. Injuries to intra-abdominal organs may be a cause of presentation. Our patient was initially asymptomatic, but later developed symptoms.

Clinically, localising the migrated pin may be challenging. Plain abdominal radiograph done (Fig. 2A and B) identified the pin as being obliquely oriented in the posterolateral aspect of the left lumbar/iliac fossa regions. The plain abdominal radiograph could not state if the pin was completely intra-abdominal. The abdominal ultrasonography was able to identify the Steinmann pin within the abdominal cavity (Fig. 3). The ultrasonography excluded visceral injury. However, it could not state if the pin was extra-peritoneal.

Laparoscopy appeared to be the gold standard as it is both diagnostic and therapeutic.

Some authors reported retrieval of sharp orthopaedic instruments [6,7] and repair of liver laceration [6]. Report of another migrated sharp instrument was documented in literature [8]. In our index patient, no visceral injury was seen. The manner of presentation, diagnosis and retrieval makes it unique and innovative. Furthermore, applying the ergonomics correctly and having adequate training with experience is the key to successful retrieval [9].

The major morbidity following open retrieval has been significantly reduced by laparoscopic retrieval. The authors encourage bending of the protruding part of pin during insertion or to use alternative pins to prevent migration.

## 7 Conclusion

Migration of Steinmann pin is unusual. Laparoscopy is an innovative approach to foreign body retrieval. This could help reduce the accompanying morbidity that may occur using other modalities such as open approach.

## Declaration of Competing Interest

No Conflict of interest.

## Source of funding

No Source of Funding.

## Ethical approval

Exemption of Ethical approval was given because no identifiable patient’s parts were seen and case was performed as emergency.

## Consent

Written and signed consent to publish as case report was obtained from the patient.

## Authors’ contribution

POI - performed the procedure, conception, drafting of the manuscript and design and have given final approval of the version to be published and agree to be accountable for all aspects of the work in ensuring that questions related to the accuracy or integrity of any part of the work are appropriately investigated and resolved.

NEB - Assisted the procedure, design and drafting the manuscript and also agree to be accountable for all aspects of the work in ensuring that questions related to the accuracy or integrity of any part of the work are appropriately investigated and resolved. Acquisition of data, or analysis and interpretation of data and drafting the manuscript.

NE - Conducted the imaging studies, acquisition of data, or analysis and interpretation of data, including interpretation of radiological images.

SLH - Acquisition of data and revising it critically for important intellectual content.

## Registration of research studies

Innovation and Case report.

## Guarantor

PO Igwe.

## Provenance and peer review

Not commissioned, externally peer-reviewed.
